# Mapping Genetic Diversity of Cherimoya (*Annona cherimola* Mill.): Application of Spatial Analysis for Conservation and Use of Plant Genetic Resources

**DOI:** 10.1371/journal.pone.0029845

**Published:** 2012-01-09

**Authors:** Maarten van Zonneveld, Xavier Scheldeman, Pilar Escribano, María A. Viruel, Patrick Van Damme, Willman Garcia, César Tapia, José Romero, Manuel Sigueñas, José I. Hormaza

**Affiliations:** 1 Bioversity International, Regional Office for the Americas, Cali, Colombia; 2 Ghent University, Faculty of Bioscience Engineering, Gent, Belgium; 3 Instituto de Hortofruticultura Subtropical y Mediterránea, (IHSM-UMA-CSIC), Estación Experimental La Mayora, Algarrobo-Costa, Málaga, Spain; 4 World Agroforestry Centre (ICRAF), GRP1 - Domestication, Nairobi, Kenya; 5 PROINPA, Oficina Regional Valle Norte, Cochabamba, Bolivia; 6 Instituto Nacional Autónomo de Investigaciones Agropecuarias (INIAP) Panamericana sur km1, Quito, Ecuador; 7 Naturaleza y Cultura Internacional (NCI), Loja, Ecuador; 8 Instituto Nacional de Innovación Agrícola (INIA), La Molina, Lima, Peru; University of Umeå, Sweden

## Abstract

There is a growing call for inventories that evaluate geographic patterns in diversity of plant genetic resources maintained on farm and in species' natural populations in order to enhance their use and conservation. Such evaluations are relevant for useful tropical and subtropical tree species, as many of these species are still undomesticated, or in incipient stages of domestication and local populations can offer yet-unknown traits of high value to further domestication. For many outcrossing species, such as most trees, inbreeding depression can be an issue, and genetic diversity is important to sustain local production. Diversity is also crucial for species to adapt to environmental changes. This paper explores the possibilities of incorporating molecular marker data into Geographic Information Systems (GIS) to allow visualization and better understanding of spatial patterns of genetic diversity as a key input to optimize conservation and use of plant genetic resources, based on a case study of cherimoya (*Annona cherimola* Mill.), a Neotropical fruit tree species. We present spatial analyses to (1) improve the understanding of spatial distribution of genetic diversity of cherimoya natural stands and cultivated trees in Ecuador, Bolivia and Peru based on microsatellite molecular markers (SSRs); and (2) formulate optimal conservation strategies by revealing priority areas for *in situ* conservation, and identifying existing diversity gaps in *ex situ* collections. We found high levels of allelic richness, locally common alleles and expected heterozygosity in cherimoya's putative centre of origin, southern Ecuador and northern Peru, whereas levels of diversity in southern Peru and especially in Bolivia were significantly lower. The application of GIS on a large microsatellite dataset allows a more detailed prioritization of areas for *in situ* conservation and targeted collection across the Andean distribution range of cherimoya than previous studies could do, i.e. at province and department level in Ecuador and Peru, respectively.

## Introduction

Many useful tropical and subtropical tree species, even those commonly cultivated, are still in incipient stages of domestication, with their genetic resources often principally or exclusively, present *in situ*, i.e. on farm in home gardens or orchards and/or in natural populations. The local diversity of these tree species could offer yet-unknown traits of high value to further domestication [1]. For many outcrossing species, such as most tropical tree species, this genetic diversity is important to sustain local production as many of these species are vulnerable to inbreeding depression [2]. Diversity is also a key factor for adaption to environmental changes [2]. However, tree species are increasingly vulnerable to losses of genetic diversity, referred to as genetic erosion, due to decreased population sizes resulting from land use changes and land degradation, and due to changes in local climate that may select against some genotypes [3]. Therefore, there is a growing call to assess the conservation status of the genetic resources of tree species [4].

The formulation of effective and efficient conservation strategies requires a thorough understanding of spatial patterns of genetic diversity [5]. A better knowledge of areas of high genetic diversity is also important in optimizing the use of genetic resources, as the likelihood to find interesting materials for breeding is higher where levels of genetic diversity are maximal [6], [7]. Initiatives to prioritize research on global plant genetic resources, such as those lead by the Food and Agriculture Organization of the United Nations (FAO), include calls for more inventories and surveys to increase understanding of variation in plant genetic resources, explicitly referring to the application of molecular tools in such assessments [8], [9].

This study focuses on cherimoya (*Annona cherimola* Mill.), an underutilized fruit tree species that belongs to the Annonaceae, a family included within the Magnoliales in the Eumagnoliid clade among the early-divergent angiosperms [10]. This Neotropical tree species still is in its initial stages of domestication [11] and it is considered at high risk of losing valuable genetic material from its genepool [12]. Cherimoya fruits are widely praised for their excellent organoleptic characteristics, and the species is therefore considered to have a high potential for commercial production and income generation for both small and large-scale producers in subtropical climates [13]. Cherimoya presents protogynous dichogamy, i.e. it has hermaphroditic flowers wherein female and male parts do not mature simultaneously, which favors outcrossing in its native range [14]. For commercial production, hand pollination with pollen and stamens is common practice due to lack in overlap of the female and male stages and absence of pollinating agents outside its native range [14]. At present, advanced commercial production is found in Spain, the world's largest cherimoya producer, with around 3000 ha of plantations, while small-scale cultivation occurs throughout the Andes, Central America and Mexico.

Most early chroniclers and scientists proposed the Andean region, and more specifically, the valleys of southern Ecuador and northern Peru, as cherimoya's centre of origin [12], [15], [16]. The existence of natural cherimoya forest patches, which are scattered across the inter-Andean valleys in Ecuador and northern Peru, supports this hypothesis. Nonetheless the possibility that these are feral populations cannot be excluded. This phenomenon has been observed in the case of several fruit tree species, such as olives [17]. An alternative hypothesis for the centre of origin of cherimoya is Central America [18], which would imply that the area of northern Peru and southern Ecuador is a secondary centre of diversity. Most relatives of cherimoya are native to Central America and southern Mexico, which is an argument in favor of this alternate hypothesis (H. Rainer, Institute of Botany, University of Vienna, 2011, pers. comm.). In any case, cherimoya fruits were consumed in the Andean region in antiquity [12] and the movement of germplasm across Mesoamerica, southern Mexico and the Andes probably took place in pre-Columbian times.

The conservation status of cherimoya genetic resources has improved considerably in recent years. Due to increasing commercial prices for cherimoya at local markets, Andean farmers are stimulated to conserve *in situ* the cherimoya trees growing in their backyards. Indeed, trees established in home gardens and orchards are common throughout the Andean region in Bolivia, Ecuador and Peru, which usually originate from planted local seeds or chance seedlings [11], and among them some individuals show promising traits for future breeding programs [19]. In Peru, the local cultivar ‘Cumbe’ is already fetching retail prices significantly above the prices of unselected cherimoya fruit types [20]. In contrast to most tropical and subtropical underutilized fruit tree species, cherimoya genetic resources are also well conserved *ex situ*. Several field collections have been established in Spain, Peru and Ecuador, preserving over 500 different accessions [11], [21]. The Spanish collection based at la Estación Experimental La Mayora in Malaga, which holds over 300 accessions (190 of them collected in the Andean region), is currently used as a source of materials for the Spanish cherimoya breeding program and has been thoroughly analyzed using isozymes [22]–[24] and microsatellite markers [11], [25]–[27].

The recent development of new molecular tools in combination with new spatial methods and increased computer capacity has created opportunities for new applications of genetic diversity analyses [28]–[30]. Whereas neutral molecular markers are considered a sound tool to measure patterns and trends in the use and conservation of plant genetic resources [31], Geographic Information Systems (GIS) provide opportunities to carry out spatial analyses of genetic diversity patterns identified with these markers [32]. GIS can be used to interpolate genetic parameters between sampled populations (e.g. [33]–[35]), to apply re-sampling of georeferenced samples within a defined buffer zone [36], [37], or to develop grid-based genetic distance models [38], [39]. GIS are also an acknowledged tool to prioritize areas for conservation of plant genetic resources [40]. Several studies have used spatial analysis to develop conservation strategies for plant genetic resources based on molecular marker data (e.g. [36], [41]). Moreover, results obtained using GIS can be presented in a clear way on maps, which facilitates the incorporation of these findings into the formulation of conservation strategies and the implementation of conservation measures [42].

In this article we further explore the possibilities of incorporating molecular marker data into GIS to better visualize and understand spatial patterns of genetic diversity, as a key input to optimize conservation and enhance use of local plant diversity, based on a case study of cherimoya. The specific objectives of this article are to (1) apply innovative spatial analysis to improve understanding of the geographic distribution of cherimoya ‘s genetic diversity in its putative native range, based on microsatellite molecular markers (SSRs); and (2) formulate optimal conservation strategies by prioritizing areas for *in situ* conservation and identifying existing diversity gaps in *ex situ* collections. Based on the outcomes, we discuss how these spatial analyses can be used to define possible strategies that guarantee the long term conservation of cherimoya genetic resources and how these analyses can be applied to improve conservation and use of tree and crop genetic resources in general.

## Results

**Figure 1:**
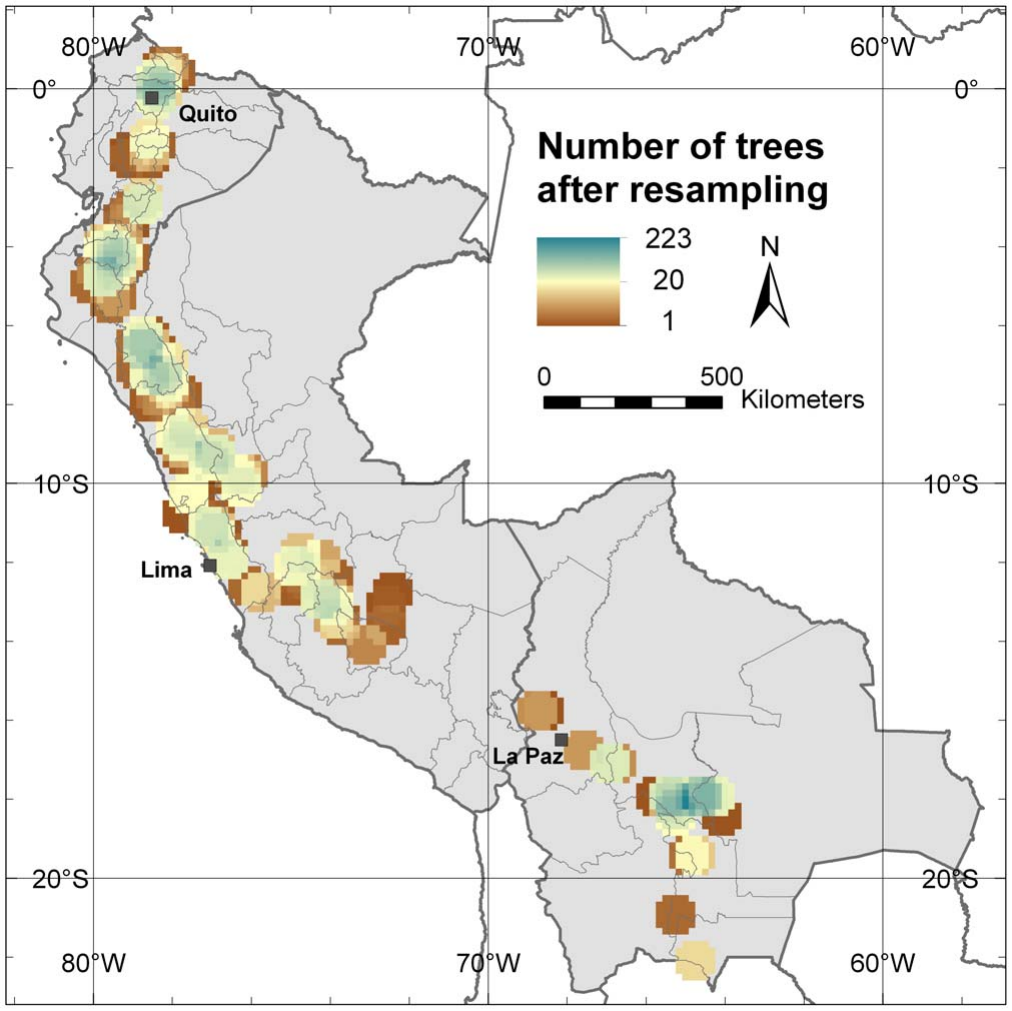
Number of trees per grid after re-sampling. This map is made with a 10-minutes grid applying a one-degree circular neighborhood.

A total of 1504 trees were analyzed in this study, i.e. 395 from Bolivia, 351 from Ecuador and 758 from Peru. Of those, 502 are currently conserved in *ex situ* collections (either in Ecuador, Peru or Spain) whereas the remainder trees were sampled *in situ*. The molecular analysis included a core set of nine microsatellite loci [27] resulting in 71 different alleles. In all analyses of *α*-diversity and *β*-diversity (also referred to as divergence) we applied circular neighborhood re-sampling technique resulting in a total dataset of 48,128 trees (Figure 1). This technique facilitates analysis of patterns in genetic variation across extensive distribution ranges while maintaining high-resolution grids.

### Allelic richness

**Figure 2:**
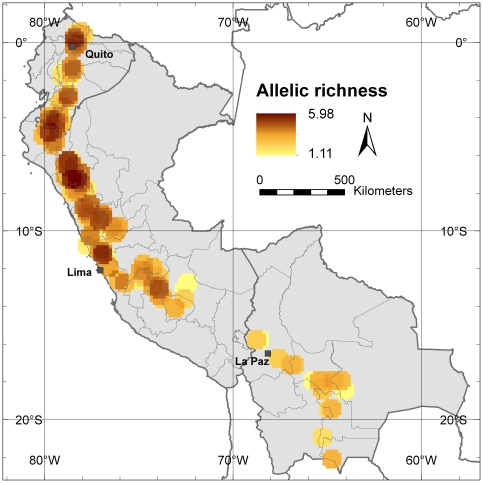
Allelic richness. This map shows the average number of alleles per locus in all 10-minutes grid cells applying a one-degree circular neighborhood.

Allelic richness is a straightforward measure of genetic diversity that is commonly used in studies based on molecular markers that aim at selecting populations for conservation [5], [43]. Figure 2 presents the distribution of the average number of alleles per locus found in the study area. It clearly shows that a higher number of alleles is present in the northern part of the study area, specifically in northern Peru, around Cajamarca Department, while other areas of high diversity are located on the border zone between Ecuador (Loja Province) and Peru (Piura Department), in the northern part of Ecuador around its capital Quito and in the northern part of the Lima Department in Peru.

**Figure 3:**
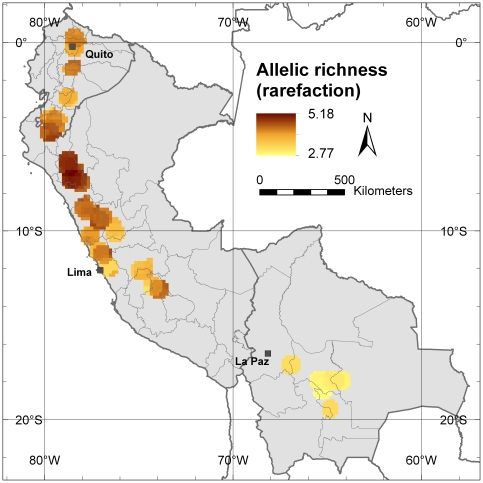
Allelic richness corrected for sample size by using rarefaction. This map shows the average number of alleles per locus in the 10-minutes grid cells applying a one-degree circular neighborhood and a correction by rarefaction to a minimum sample size of 20 trees.

Despite the effort to implement a similar sampling density throughout the study area, some areas (often locations with a higher abundance of traditionally managed cherimoya trees and stands) have been sampled more intensively than others (Figure 1), generating a sampling bias [44]. The rarefaction methodology corrects this sampling bias by recalculating allelic richness in each grid cell to a minimum sample size [5]. Figure 3 shows only the grid cells where 20 or more trees were present after applying a one-degree circular neighborhood, and for which allelic richness was corrected following the rarefaction methodology to a minimum sample size of 20 trees. The Cajamarca Department in northern Peru remains the area with the highest diversity, up to an average of 5.18 different alleles per locus. After correction by rarefaction, diversity in Ecuador, especially around Quito, is reduced, whereas the same seems to happen in the northern part of the Lima Department, in Peru, indicating the presence of a sampling bias around the capitals of both countries. The area around the Peruvian capital Lima, an important commercial cherimoya cultivation area, shows the lowest allelic richness within Peru, probably due to the widespread cultivation of a vegetatively propagated cultivar, ‘Cumbe’. Another striking result is that allelic richness in Bolivia, already low in the uncorrected analysis, is even lower with correction of sampling bias, resulting in an even higher contrast between cherimoya genetic diversity in Bolivia and that found in Peru and Ecuador.

### Locally common alleles

**Figure 4:**
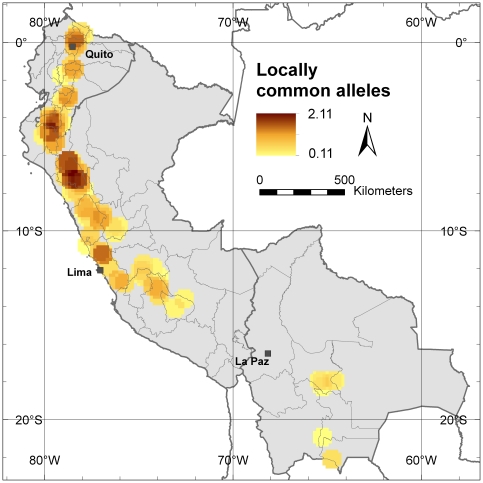
Locally common alleles. This map shows the average number of alleles per locus in a 10-minutes grid cell that are relatively common (occurring with a frequency higher that 5%) in a limited area (in 25% or less of the grid cells) applying a one-degree circular neighborhood.

Priority for conservation should be given to populations that retain locally common alleles; these are alleles that occur in high frequency in a limited area, and can indicate the presence of genotypes adapted to specific environments [43]. Figure 4 shows the richness of locally common alleles per locus in the study area. The high diversity levels found in the Cajamarca Department in northern Peru are reconfirmed. Besides harboring the highest number of different alleles, it also contains the highest number of locally common alleles. This makes this area a priority for *in situ* conservation, both of cultivated trees on farm and of natural stands. The border region between Peru and Ecuador (Piura Department and Loja Province) is another area where a high concentration of locally common alleles has been observed and may, therefore, be a second area to prioritize *in situ* conservation efforts. To a lesser extent, the area around Quito in Ecuador and the northern part of the Lima Department in Peru also present locally common alleles.

### Expected Heterozygosity (He), Fixation Index (F) and Genetic Distance (GD)

**Figure 5:**
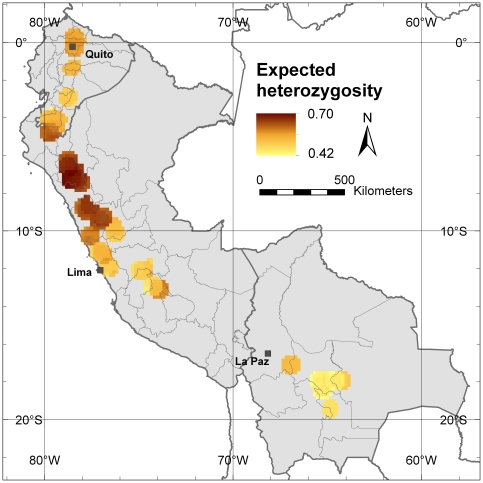
Expected heterozygosity (*He*). This map shows the average *He* value in each 10-minutes grid cell with 20 or more trees applying a one-degree circular neighborhood.

**Figure 6:**
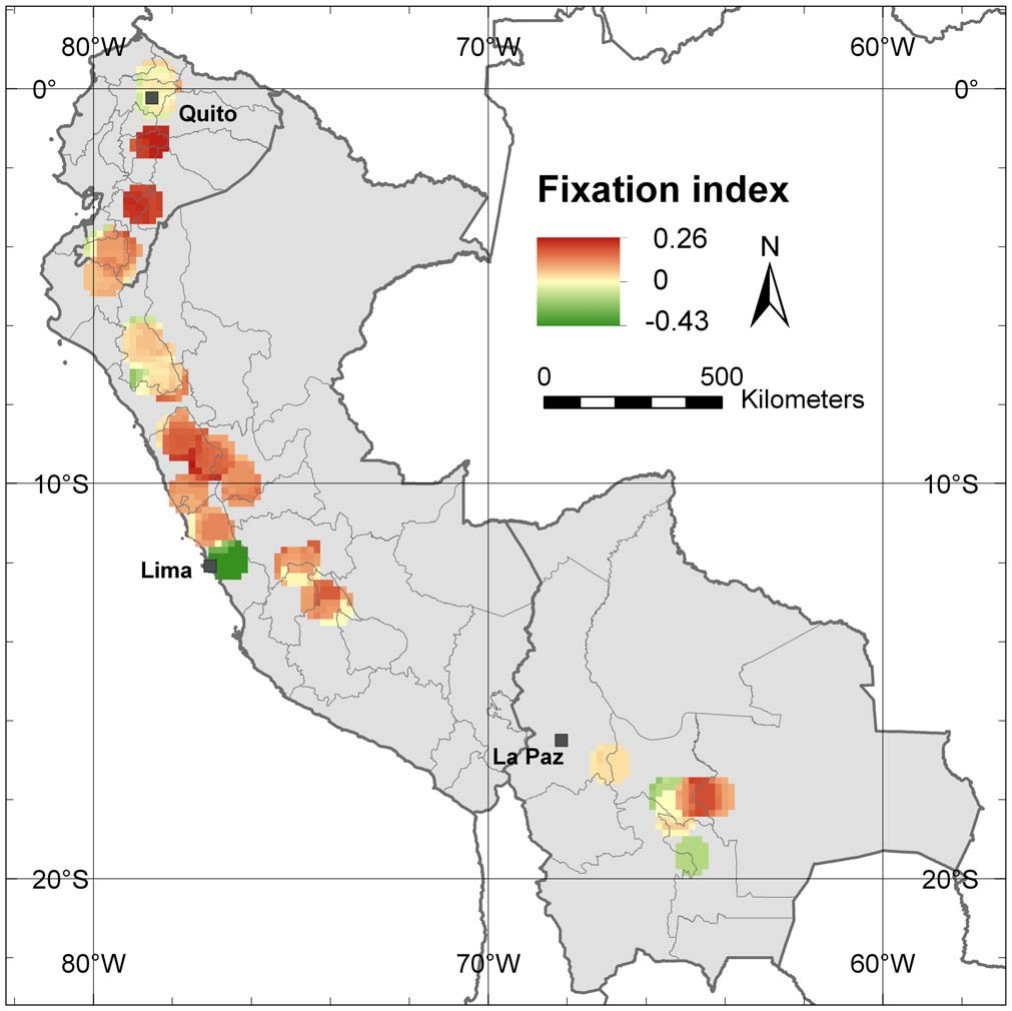
Fixation *index (F)*. This map shows the average *F* value in each 10-minutes cell with 20 or more trees applying a one-degree circular neighborhood. Yellow areas indicate cherimoya stands where observed heterozygosity is as expected, red areas indicate stands where observed heterozygosity is lower than expected (indicating inbreeding) whereas observed heterozygosity is higher than expected in green areas.

*In situ* conservation should focus on viable populations, where inbreeding and subsequent loss of alleles are minimal. Parameters that allow assessment of inbreeding are expected heterozygosity (*He*) and the fixation index (*F*). The fixation index (*F*) was used to detect areas subjected to high inbreeding depression and, as the inverse to that, excess in heterozygosity [45]. Figure 5 shows the values for *He* in the study area, again confirming Cajamarca Department in northern Peru as the area with the highest genetic diversity. High *He* values, however, radiate towards the south (as opposed to the higher diversity towards the north found in the allelic richness analyses) indicating higher levels of diversity in terms of heterozygosity in central Peru compared to Ecuador. Figure 6 shows the values for the fixation index, with *F* values close to 0 in the Cajamarca Department indicating that natural and cultivated cherimoya tree stands in this area have not experienced inbreeding. The highest values for *F* are observed in central Ecuador, suggesting that the level of inbreeding is highest in that part of cherimoya's Andean distribution range.

**Figure 7:**
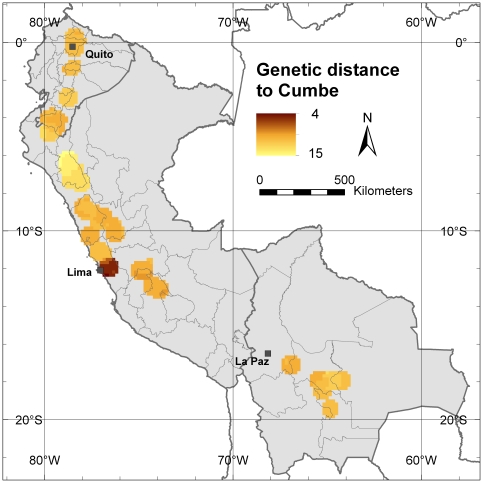
Genetic distance to the Peruvian cultivar ‘Cumbe’. This maps shows the average genetic distance (GD) to the cultivar ‘Cumbe’, in each 10-minutes cell with 20 or more trees applying a one-degree circular neighborhood. As reference of the cultivar, the ‘Cumbe’ accession from the collection la Mayora, Malaga, Spain, was used.

The most important Peruvian commercial cherimoya cultivation area, located near the Capital Lima, particularly shows negative *F* values, i.e. excess of heterozygosity. Most of the cherimoyas cultivated in this area are vegetatively propagated clones of the cultivar ‘Cumbe’ which resulted in highly heterozygous values from the molecular analysis, i.e. the ‘Cumbe’ accession conserved in the Spanish genebank is heterozygote for eight of the nine microsatellite loci analyzed in this study (*Ho* value of 0.89). An analysis of the average genetic distance, between the ‘Cumbe’ accession and the genotypes in each grid cell with 20 or more re-sampled trees in the study area, clearly shows lowest genetic distance values near the Peruvian capital, Lima, indicating that the cherimoya trees in this area are very similar to the cultivar ‘Cumbe’ (Figure 7). This area clearly differs from the rest of the cherimoya distribution area in our study, which is more likely to be a product of natural gene flow patterns.

### β-diversity (divergence)

**Figure 8:**
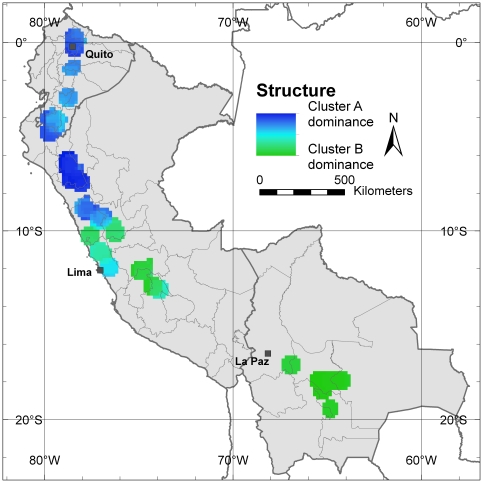
Genetic structure of Andean cherimoya distribution in Population clusters A and B. This map shows in each 10-minutes cell with 20 or more trees applying a one-degree circular neighborhood, the average probability of finding a cherimoya tree belonging to cluster A or B. Dark blue areas show a higher probability of finding trees belonging to cluster A whereas dark green areas show a higher probability of finding trees belonging to cluster B. Light blue colored areas are not clearly assigned to any of the two clusters.

Besides *α*-diversity parameters, aimed at identifying those areas with highest allelic richness and balanced allele frequencies, *in situ* conservation also needs to take into account allelic composition (*β*-diversity or divergence) as it is possible that populations with low allelic richness possess unique allele compositions, different from those of populations in other areas of the range, which would warrant their *in situ* conservation [5]. Applying the Structure software (see [46]) and using the statistic parameter ΔK [47] to define the number of clusters with genetically similar trees present in the study area, we differentiated two main populations. Figure 8 shows the differentiation of the populations among distribution areas in cluster A and B, respectively. Cluster A has the highest presence in the areas previously identified as those with the highest allelic richness (Cajamarca Department in northern Peru, border zone between Ecuador and Peru and the area around Quito in Ecuador), whereas cluster B is mainly confined to southern Peru and Bolivia. Bolivian cherimoya trees are almost exclusively assigned to cluster B. Particular areas that did not show a strong linkage to either of the two clusters included the surroundings of the city of Lima and Loja Province in southern Ecuador.

### *Ex situ* conservation status

**Figure 9:**
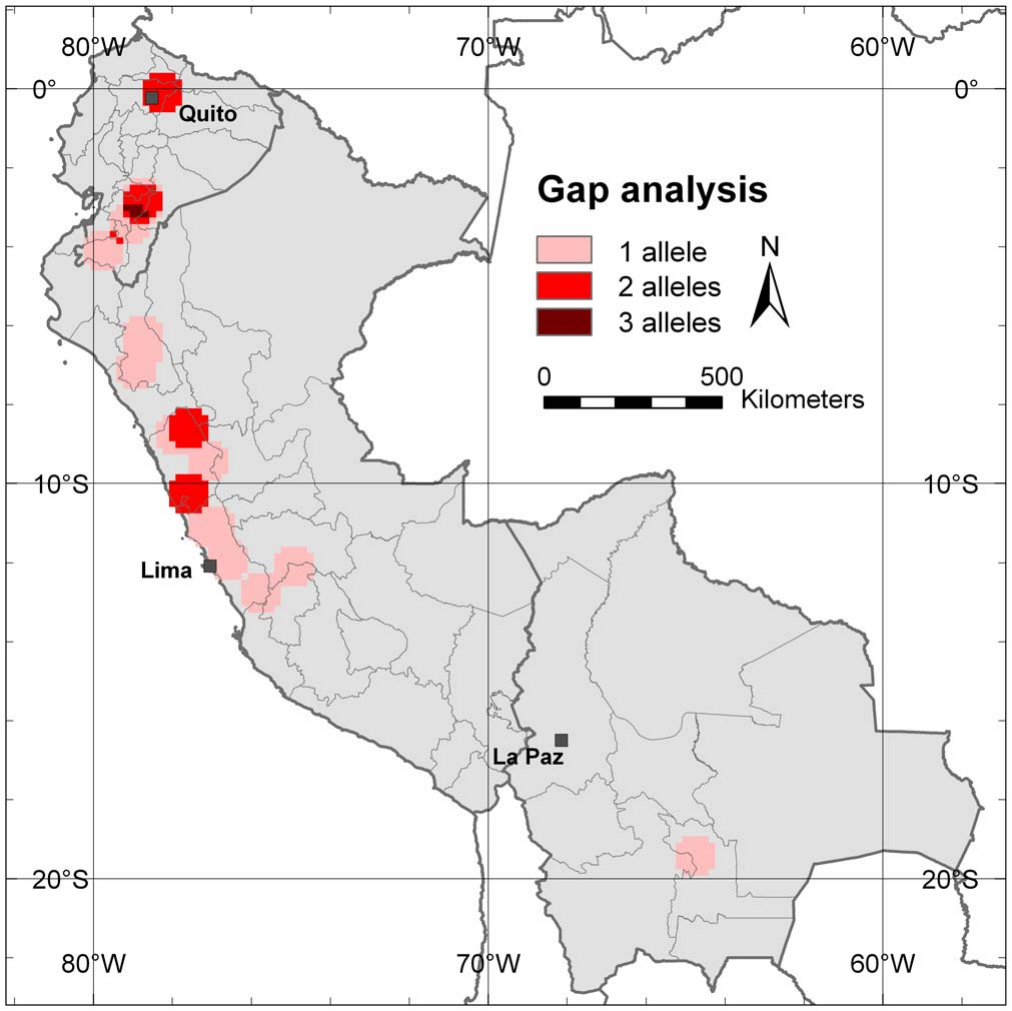
Gap analysis of alleles not found in *ex situ* collections. Richness analysis of alleles (eight alleles out of the total of 71 observed alleles) that are not found in any *ex situ* collection based on 10-minutes grid with a one-degree circular neighborhood.

Of the 1504 trees included in this study, 502 genotypes are currently conserved in *ex situ* collections (either in Ecuador, Peru or Spain). Only eight alleles, corresponding to 11% of the total of 71 alleles that have been found in the study area, are not represented in any accession of these collections. Figure 9 shows the distribution of the missing alleles. There is only a small area with a significant portion of missing alleles (3 in total), i.e. in southern Ecuador (Azuay Province). Natural cherimoya forest patches and areas of traditional cherimoya cultivation in this province should be prioritized for future cherimoya collection missions. With almost 90% of alleles found to be present in *ex situ* collections, it can be concluded that, in general, cherimoya diversity from the countries analyzed is fairly well conserved *ex situ*.

### Distribution range of cherimoya in the Andes

The above results and subsequent conclusions are obviously only of practical use if the sampling performed was indeed representative for the distribution of cherimoya in the study area. Maxent species distribution modeling software was applied to model cherimoya's distribution range in Ecuador, Peru and Bolivia based on the climatic niche in which the 1504 sampled trees of our study were located. The modeled distribution was then compared with the sampled areas in these countries.

Cross-validation, to evaluate the quality of the distribution model, returned an Area Under Curve (AUC) value of 0.9, which indicates good model performance [48]. AUC is a commonly used parameter in the validation of distribution models. Another measure of validation, the Kappa value, returned a value of 0.799 indicating the model performed even excellent [49].

**Figure 10:**
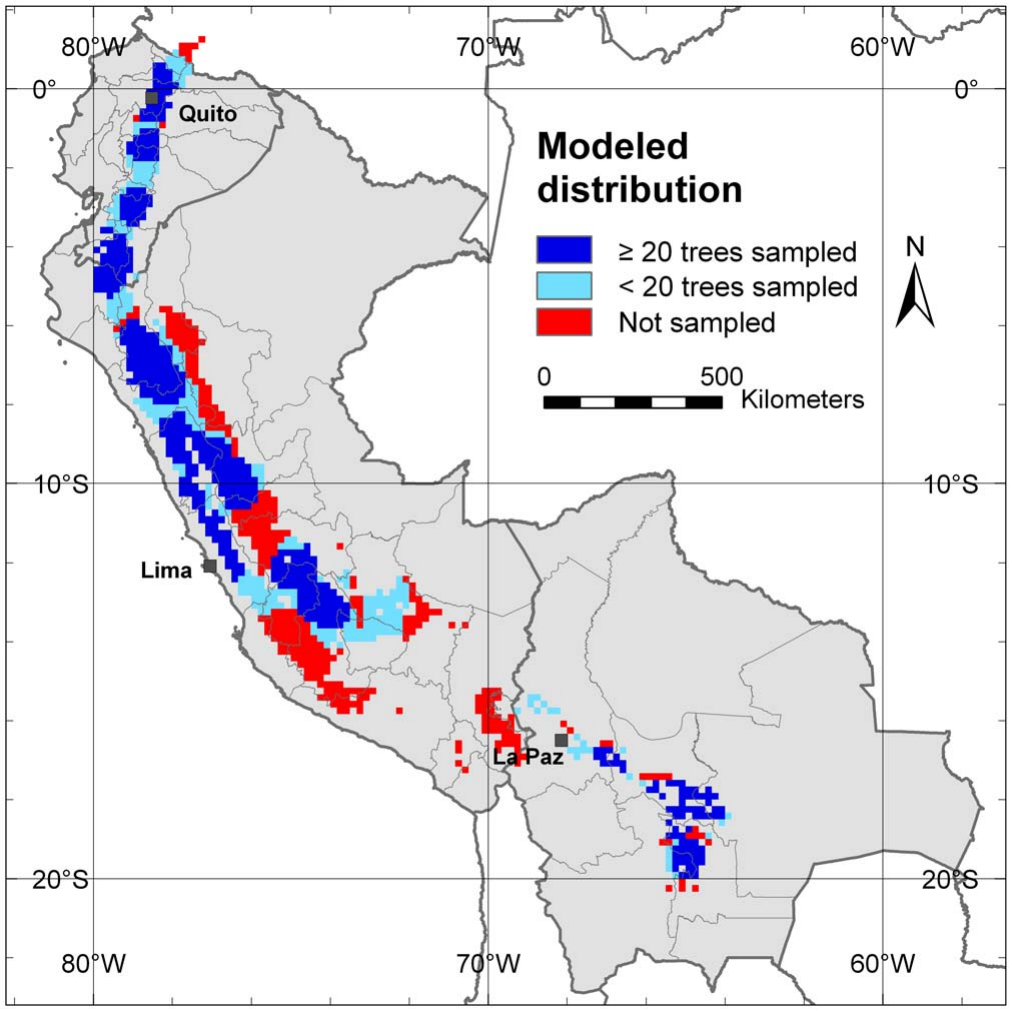
Modeled distribution of cherimoya. Areas of the modeled distribution in dark blue are covered by the 10-minutes grid cells with 20 or more trees applying circular neighborhood. Light blue areas of modeled distribution coincide with grid cells that contain less than 20 trees after re-sampling. Red areas indicate potential areas for cherimoya occurrence and cultivation that have not been in sampled.

In general, sampling covered most of the cherimoya-modeled distribution (Figure 10); 46% of the modeled distribution area is covered by grid cells with 20 or more re-sampled trees (Figure 10, dark blue areas). In 24.5% of the potential area of cherimoya occurrence less than 20 trees were re-sampled (light blue areas) whereas 29.5% of the modeled range was not sampled (red areas) and are considered sample gaps. The largest sample gaps are located in northern Peru in the transition zone between the Peruvian Andes and the Amazon (in the Departments of San Martin and Amazonas) and in southern Peru (in the Departments of Junín, Pasco, Huancavelica, Ayacucho and Puno). The Andean-Amazon transition zone should be priority for future complementary cherimoya collection trips because it is adjacent to an area where already high levels of diversity have been found, i.e. Cajamarca Department in northern Peru.

Cherimoya was predicted absent by the distribution model in a significant area of southern Peru, indicating that the environmental conditions in substantial parts of that region are not suitable for cherimoya cultivation (Figure 10). This explains why no trees have been sampled in that area.

## Discussion

### Areas of high diversity in the cherimoya centre of origin

Our results are in line with a previous genetic study of the Spanish cherimoya collection that also distinguished populations in Ecuador and northern Peru from those in southern Peru [11], and corroborate with results from isozyme markers that showed high genetic variation present in Peru and Ecuador [24]. However, our study is based on a much higher number of samples and, therefore, provides much more detail for prioritizing areas for *in situ* conservation and germplasm collection.

At the allele level, our analysis confirms that, within our study area, the highest allelic richness as well as the highest number of locally common alleles are found in the area of southern Ecuador and northern Peru, i.e. the putative centre of origin of cherimoya. Northern Peru, and more specifically the Cajamarca Department, shows the highest levels of genetic diversity.

The highest values of the fixation index, which is an indication of inbreeding, were found in Ecuador. Inbreeding may occur because of reduction and fragmentation of natural stands and cultivated areas, increasing the risk of allele loss, which eventually leads to genetic erosion [50]. Our results do not allow us to determine how much genetic erosion has taken place in Ecuador in comparison to Peru and Bolivia, but the high inbreeding values in Ecuador could explain why currently allelic richness is lower in this country than in northern Peru.

At the population level, significant differences can be observed between the cherimoya germplasm present in the area with highest diversity (where genotypes belonging to cluster A are predominant) and genotypes found in areas with lower diversity, i.e. in southern Peru and Bolivia (represented by cluster B). Cluster A seems likely to represent material that is genetically closer to the “wild” cherimoya type. No natural cherimoya stands have been observed in Bolivia, and this probably explains why no genotypes pertaining to cluster A have been recorded there. Cluster B probably corresponds to a genepool that is genetically different from most of the wild or semi-domesticated cherimoya found in northern Peru and Ecuador and that could have formed the basis for Bolivian cherimoya cultivation. Looking at the areas of high cluster B dominance, Bolivian germplasm probably originates from southern Peru.

Although most early chroniclers and scientists proposed southern Ecuador and northern Peru to be cherimoya's centre of origin [12], [15], [16], [51], the possibility of that area being a secondary centre of origin cannot be discarded. A diversity study similar to the one described in this study, but including cherimoya genotypes from Central America and Mexico, would shed light on the genetic variation across the complete pre-Columbian distribution range of cherimoya and provide additional clues on the primary centre of origin and diversification of this species.

### *Ex situ* and *in situ* conservation of cherimoya genetic resources in the Andean region

Most alleles identified in our study are represented in one or more of the existing *ex situ* collections in Ecuador, Peru and Spain. The results obtained suggest that the highest priority for further collection should be the Azuay Province in Ecuador, since cherimoya stands in this area harbor most alleles not yet included in genebanks. It is also one of the areas with the highest risk of allele loss because of the high observed levels of inbreeding, compared to other parts of the study area. An additional priority area for germplasm collection is the transition zone from the Andes to the Amazon in Peru (in the higher elevation areas of the Departments of San Martin and Amazonas), which was not sampled in this study. According to the distribution model there is a high probability of finding cherimoya stands in this region, which probably is also high in genetic diversity, because it is adjacent to the area with the highest diversity found in this study, i.e. the Cajamarca Department in northern Peru.

A priority for *in situ* conservation should be the Cajamarca Department, the area with the highest levels of genetic diversity. A second area of priority should be the Loja Province in southern Ecuador, an area with a high number of locally common alleles. Both areas are assigned mostly to cluster A. Since trees pre-dominantly assigned to cluster B have a particular allelic composition in comparison to trees predominantly grouped in cluster A, genotypes of cluster B should also be considered in conservation activities. The part of Lima Department north of the Peruvian capital, which is assigned mostly to cluster B, could be prioritized for *in situ* conservation of genotypes from this cluster. In contrast to the low levels of allelic richness around Lima city in the southern part of the Lima Department, the northern part of this Department contains a fair number of locally common alleles.

The long-term conservation of cherimoya genetic resources is far from guaranteed. As commercial prices for fruits can fluctuate, short-term incentives for farmers to maintain cherimoya as a profitable crop are reduced and a decline in commercial interest may lead to the replacement of cherimoya trees by other crops, increasing the risks of genetic erosion. Around Quito, for example, most of the traditional cherimoya cultivation is being replaced by avocado plantations, which are commercially more attractive (X. Scheldeman, pers. obs.). An increase in commercial prices for cherimoya products will not necessarily promote the conservation of the existing genetic diversity. Indeed, in our study we found low levels of genetic diversity around the Peruvian capital, Lima where the clonally propagated cultivar ‘Cumbe’ is widely cultivated, because it fetches higher prices in the market.

A promising strategy to enhance *in situ* conservation on farm is through the promotion of seed or bud-for-grafting exchange between farmers [52]. During the CHERLA project, cherimoya fairs, which facilitate exchange of plant material, were organized in different areas of this study, including the Cajamarca and Piura Departments in Peru, Loja Province in Ecuador and various departments in Bolivia. Seed and bud exchange can also be a way to conserve local races from unfavorable alterations in the local environment due to climate change, by re-distributing them in new areas with suitable climate conditions [53]. Another way to ensure conservation of genetic resources of tree species while their use is stimulated could be the establishment of local clonal seed orchards if and when adequate propagation techniques, to enable the multiplication of clones, are made available as well [1], [54]. This is the case for cherimoya, as demonstrated by the successful clonal propagation of the cultivar ‘Cumbe’ around the city of Lima.

Ideally, each area targeted for *in situ* conservation - where the existing cherimoya stands and forest patches can evolve within the local environment - should be backed up by *ex situ* conservation of germplasm (which currently is the case for cherimoya), and be monitored periodically to assess the dynamics in diversity use and risks of genetic erosion. *Ex situ* collections of fruit tree species often consist of living trees, such as the cherimoya collections. This allows conservation of superior combination of alleles that can be propagated vegetatively through grafting. Additional reasons include the following: (1) many tropical and subtropical trees (including cherimoya) have seeds with recalcitrant or intermediate behavior, which cannot be stored for long-term conservation; and (2) pollen, fruits and seeds can be collected continuously for characterization, evaluation and genetic improvement once trees have reached the reproductive stage. Nevertheless, the high costs for research institutions to maintain field genebanks of woody perennial species, can be a reason to minimize *ex situ* collections and focus especially on *in situ* conservation [55]. In that case, it is important to screen the existing accessions through morphological, biochemical and/or molecular characterization to maximize the conservation of genetic diversity and potentially interesting functional attributes in a reduced collection [6]. This approach has already successfully been used in the cherimoya collection la Mayora, Malaga, Spain [27]. *Ex situ* conservation may particularly be important for areas that suffer from inbreeding -an indicator for high rates of allelic loss and genetic erosion- such as central Ecuador in the case of cherimoya, whereas *in situ* conservation may be most successful in areas of high diversity where still low rates of inbreeding are observed such as in the cherimoya stands from northern Peru.

### Use of GIS and molecular markers to enhance conservation and use of plant genetic resources

Despite the advances in new computational applications and the use of molecular tools, spatial analyses are still underutilized in efforts to conserve plant diversity [56]. With respect to targeting collection sites and prioritizing the conservation of plant genetic resources, spatial analyses of diversity have been carried out mainly at the species level for crop genepools (e.g. [57]–[59]). Only a few studies have mapped intraspecific diversity to enhance the conservation of genetic resources of specific crops and trees (e.g. [36], [41]). Kiambi et al. [41] grouped samples using a grid to compare diversity between geographic areas of similar size, whereas Lowe et al. [36] applied re-sampling to enable the calculation of diversity estimates with high degrees of confidence. However, these studies were carried out with fewer than 100 individuals per species, which limits the type of spatial analysis that can be carried out over the geographic distribution range of species. Our analysis combines both techniques on a large dataset (1504 trees), which can be conceptualized as a continuous distribution of plant individuals, in which each individual is connected to its neighboring trees because they share the same seed system, and/or breed with each other. Based on this concept, trees have been sampled in this study following a scattered distribution to calculate, across the Andean distribution range of cherimoya, several diversity estimates important to prioritize areas for conservation, including two recommended parameters: allelic richness [5] and the number of locally common alleles [43]. Since the application of molecular tools is becoming cheaper, intraspecific diversity studies with large datasets will probably be more common in the near future, allowing for studies of this sort on other tree species and annual crops.

The size of the grid cells and width of the circular neighborhood for this type of spatial analysis depends on how many plant individuals have been collected across the landscape, and the minimum number of plant individuals that is considered sufficient to make confident estimates of genetic parameters per grid cell. Application of circular neighborhood provides an effective way to decrease grid cell size, which facilitates detection of spatial patterns in genetic variation across an extensive distribution range. By re-sampling the trees in the landscape, it generates a high number of grid cells with a sufficient number of trees to make confident calculations of genetic parameters per grid cell. It also makes analyses less sensitive to grid origin definition and enables the inclusion of isolated trees in the calculation of the genetic parameters, i.e. together with their closest neighboring trees.

Ideally, the sampling strategy for this type of analysis should be identified based on a pre-defined grid, aiming at measuring the same number trees per grid cell. However, due to logistical constraints and because a species simply may be more abundant in some areas than in others, in practice, sampling will always be sub-optimal to a certain degree. Of all the genetic parameters, allelic richness is most sensitive to uneven sampling and, accordingly, we have corrected sample size by rarefaction [5]. Repeated subsampling of a minimum number of tree individuals per grid cell is another possibility to correct for sampling bias [60]. This technique could also be used to correct other genetic parameters than allelic richness for sampling bias, such as expected heterozygosity, although these are less sensitive to uneven sampling [61].

Given the sampling distribution in our study area and the fact that for the calculation of most genetic parameters, we maintained a minimum of 20 re-sampled trees per grid cell, we defined a cell size of 10 minutes and a circular neighborhood with a diameter of one degree, which enabled us to detect spatial patterns of genetic variation at administrative level one in Ecuador, Peru and Bolivia (provinces and departments). For studies of plant species, in which individuals are sampled in a more clumped distribution compared to our scattered sampling distribution and/or in lower densities across the landscape, larger grid cells and/or a larger width of circular neighborhood could be applied, always assuring a sufficient number of trees per grid cell. The overall resolution of the study will obviously be lower.

Following Frankel et al. [6], we hypothesized that areas with high diversity measured by neutral molecular markers (like our microsatellite loci) have a high probability to contain genetic material that will also show diversity in functional traits, including traits of agronomic interest. Molecular markers are considered an appropriate indicator to quantify patterns and trends in the use and conservation of plant genetic resources [31]. However, while neutral molecular marker surveys are suitable for diversity studies, direct measurement of traits in field trials may be more desirable to evaluate the genetic health and adaptive capacity of tree populations [50]. Nevertheless, molecular marker studies representative of the whole genome provide a less expensive and scientifically sound alternative to assess the genetic resource status of tree species, for which, in comparison to annual crops, field trials are particularly expensive because of the long generation times [62]. Markers of DNA sequences related to phenotypic traits, including expressed sequence tagged (EST) markers and markers in specific genes, could be of interest to include in spatial analysis of patterns and trends in plant genetic resources. More and more are becoming available, especially for important crops where sequencing programs have been performed or will be carried out in the near future. An example in cherimoya is a recently described gene involved in seedlessness in a sister species, *Annona squamosa* [63]. However these markers are less polymorphic than neutral ones, such as those that have been used in our study, so the use of neutral markers to study spatial patterns of genetic diversity is still necessary.

It is difficult to compare our results with those of Lowe et al. [36] and Kiambi et al. [41] because of the differences in methodology used. To examine molecular marker studies on the same species, minimum standard sets of markers have already been suggested [64]. Standardization of methodologies in studies on different species would improve comparability of results and also would facilitate Meta-analyses, for example to better understand how well genetic diversity of tropical and subtropical tree species is protected on farm and in protected areas.

In our study we only examined spatial patterns of genetic variation without relating them to other spatial attributes. GIS can also be used to link genetic data to available spatial information relevant to conservation of plant genetic resources, for instance to reveal short-term threats such as accessibility and long-term threats such as climate change. With this type of analysis, hotspots of diversity under threat could be identified following Myers et al. [65] but instead of looking at species level, this could be done at the intraspecific level, to ensure the conservation of priority populations of specific crops and useful tree species. Spatial information on the patterns and characteristics of human societies can be used to understand the drivers behind threats. In a study on changes in cassava diversity in the Peruvian Amazon, GIS was used to correlate cassava diversity data with biotic and socio-economic spatial data to identify possible drivers behind diversity and genetic erosion [66]. This can be useful information in the development of adequate policies and measures to promote *in situ* conservation of plant genetic resources on farms and in natural populations.

## Materials and Methods

**Sampling and SSR analysis:** A total of 1504 cherimoya accessions have been analyzed in this study, 395 from Bolivia, 351 from Ecuador and 758 from Peru. DNA was extracted from young leaves after [67]. Based on polymorphism, a set of nine SSRs has been selected from those previously developed in cherimoya [26]. A 15 µL of reaction solution containing 16 mM (NH4)2SO4, 67 mM Tris-ClH pH 8.8, 0.01% Tween20, 2 mM MgCl2, 0.1 mM each dNTP, 0.4 µM each primer, 25 ng genomic DNA and 0.5 units of BioTaq™ DNA polymerase (Bioline, London, UK) was used for amplification on an I-cycler (Bio-Rad Laboratories, Hercules, CA, USA) thermocycler using the following temperature profile: an initial step of 1 min at 94°C, 35 cycles of 30 s at 94°C, 30 s at 45°C–55°C and 1 min at 72°C, and a final step of 5 min at 72°C. Forward primers were labeled with a fluorescent dye on the 5′ end. The PCR products were analyzed by capillary electrophoresis in a CEQ™ 8000 capillary DNA analysis system (Beckman Coulter, Fullerton, CA, USA). Samples were denaturalized at 90°C during 120 s, injected at 2.0 kV, 30 s and separated at 6.0 kV during 35 min. Each reaction was repeated twice and the Spanish cultivar Fino de Jete was used as control in each run to ensure size accuracy and to minimize run-to-run variation.

**Data cleaning:** The coordinates of the respective tree locations were checked in DIVA-GIS (www.diva-gis.org) on erroneous points based on passport data at administrative level one (e.g. departments, provinces) with a buffer of 20 minutes (approx 30 km), and outliers based on climate data derived from the Worldclim data set [68] (two or more of the 19 bioclim variables according the Reverse jackknife method [69]). Based on these analyses, two points were excluded. The cleaned dataset thus included microsatellite data of 1504 georeferenced trees. Taking into account that nine SSR markers were analyzed, this results in a total of 27,072 georeferenced alleles.

**Spatial analysis – Circular neighborhood:** Grids for all genetic parameters were generated in DIVA-GIS and are based on a grid with a cell size of 10 minutes (which corresponds to approximate 18 km in the study area) applying a circular neighborhood with a diameter of one degree (corresponding to approximate 111 km) constructed in Excel. The circular neighborhood is used to re-sample the allelic composition of a single tree to all surrounding grid cells, in this case, 32 cells with a size of 10 minutes, within a diameter of one degree around its location. In this way, the allelic composition of each sampled tree is representative for the area within the defined buffer zone. Applying the circular neighborhood re-sampling technique resulted in a total dataset of 48,128 trees and 866,304 alleles.

**Spatial analysis –** ***α*** **-diversity:** After applying circular neighborhood to all trees, genetic parameters were calculated in GenAlEx per 10-minutes grid cell, for all trees present in each cell after re-sampling. Genetic parameters included the average number of alleles per locus (*Na*), the number of locally common alleles per locus (alleles occurring with a frequency higher than 5% in 25% or less of the grid cells), average expected heterozygosity per locus (*He*), fixation index (*F*) and genetic distance (*GD*) (see [45]). *Na* and the number of locally common alleles per locus were presented for all grid cells with trees included. *Na* was corrected by rarefaction to a minimum sample size of 20 trees per cell with the HP-RARE software (see [70]); consequently, this parameter was only calculated for grid cells with 20 or more re-sampled trees. This minimum sample size was also used as a threshold of the number of trees per grid cell to get interpretable results for the parameters *He*, *F* and *GD*. *GD*, which was used to calculate distance in allelic composition of each cherimoya genotype to the commercial variety ‘Cumbe’, was calculated in GenAlEx using the *GD* option for codominant markers (see [71]). Final *GD* value per grid cell was the average *GD* for all re-sampled trees present in each cell. The reference tree was the accession ‘Cumbe’ from the Spanish cherimoya genebank in Malaga.

**Spatial analysis -** ***β*** **-diversity:** Population structure was defined by running the software Structure (see [46]) on all 1504 samples applying a 10,000 burn-in period, 10,000 Markov chain Monte Carlo (MCMC) repetitions after burn-in, and 20 iterations. Optimal K was selected after [47] by running Structure for K values between one and 10 and defining the final number of clusters where value of ΔK was highest. This was at K = 2, hence a map was developed for these two clusters, which we named respectively A and B. We used the probabilities of each tree belonging to cluster A and B to visualize the clusters on a map. Mapping of probabilities was done based on the average value of all trees per 10-minutes cell for those grid cells with 20 or more re-sampled trees after applying the one-degree circular neighborhood.

**Spatial analysis -** ***Ex situ*** **conservation status:** The private alleles function in GenAlEx (PAS) was used to identify the alleles exclusively found in trees that were sampled *in situ*. To visualize patterns in these alleles that are not included in any genebank, a point-to-grid richness analysis, using a 10-minutes grid, was carried out in DIVA-GIS based on the one-degree circular neighborhood re-sampled tree grid.

**Spatial analysis - distribution modeling:** To identify how well the sampling covered the Andean distribution range of cherimoya, and thus to identify potential collection gaps, we modeled the distribution (presence only) of cherimoya in the study area using the distribution modeling program Maxent (see [72], [73]). With this technique, potential distribution areas are identified as those areas where similar environmental conditions prevail as those at the sites where the species has already been observed. The data required to identify these areas include species presence points as well as layers of environmental variables covering the study area. Maxent is a species distribution modeling tool for which the applied algorithm has been evaluated as performing very well, in comparison to other ecological niche modeling software [74], [75]. Therefore, it was selected for this study's distribution modeling analysis. The coordinates in the passport data of the sampled trees were used for the presence point input. For environmental layer input, we used the 10-minutes grids of 19 bioclimatic variables (see [76]), derived from the Worldclim dataset [68]. The modeled distribution area was restricted using the 10 percentile training presence threshold, which indicates the probability value at which 10% of the presence points falls outsides the potential area. The modeled distribution was generated in Maxent with 80% of the points (training data) and was cross-validated in DIVA-GIS with 20% of the remaining tree observations (test data). Besides 20% of the presence points, test data included randomly generated points in 0.1× the bounding box of the presence points as a proxy for absence points (5 times the number of presence points). Based on the cross-validation, the Area Under Curve (AUC) and Kappa value were calculated in DIVA-GIS as measures of model performance.

All maps were edited in ArcMap.
